# Supplementary material for: Characterization of two affinity matured Anti-Yersinia pestis F1 human antibodies with medical countermeasure potential
Source: PLoS One. 2024 Jul 2;19(7):e0305034. doi: 10.1371/journal.pone.0305034 (PMC11218954; doi:10.1371/journal.pone.0305034)
Supplement: S1 Fig — The yeast-associated fluorescence after incubation with biotinylated F1V and staining with streptavidin-APC (allophycocyanin) was measured by flow cytometry. Mean fluorescence intensity values at antigen concentrations of 500, 50, and 5 nM are shown for the original (αF1sc 2 and 8) and the progressively affinity matured antibody clones. The nomenclature EPX-Y denotes the error-prone library number (X) and the best clone number (Y) identified from this library. The best affinity matured αF1sc 2 clone (indicated for simplicity as αF1sc AM2) was clone 18 from error-prone library 3 (αF1sc 2 EP3-18). The best affinity matured αF1sc 8 clone (indicated as αF1sc AM8 for simplicity) was clone 24 from error-prone library 2 (αF1sc 8 EP2-24). (PDF) [file pone.0305034.s001.pdf]

A

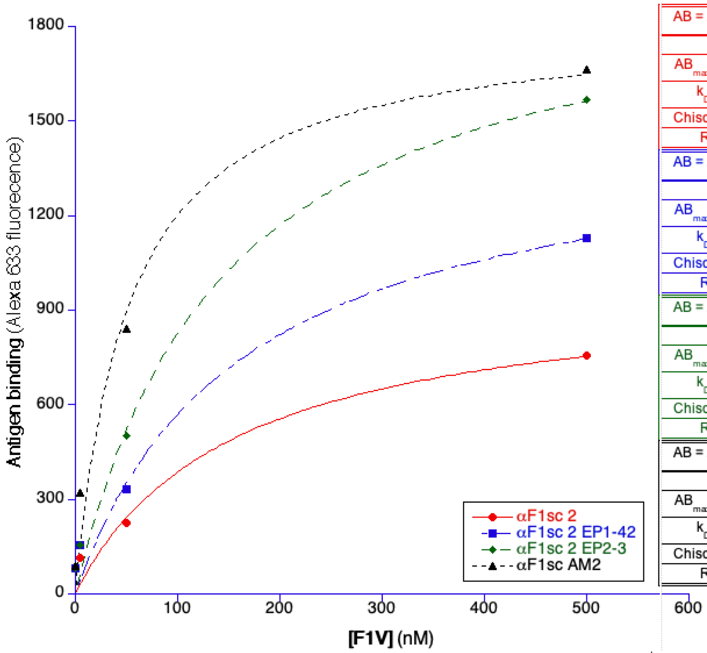

AB = AB$_{max}$*[Ag]/(k$_D$+[Ag])		
	Value	Error
AB$_{max}$	986.4	225.99
k$_D$	154.48	109.33
Chisq	14698	NA
R	0.97455	NA

AB = AB$_{max}$*[Ag]/(k$_D$+[Ag])		
	Value	Error
AB$_{max}$	1491.8	269.32
k$_D$	162.28	89.277
Chisq	18910	NA
R	0.98629	NA

AB = AB$_{max}$*[Ag]/(k$_D$+[Ag])		
	Value	Error
AB$_{max}$	2018.1	213.29
k$_D$	144.62	48.025
Chisq	14841	NA
R	0.99472	NA

AB = AB$_{max}$*[Ag]/(k$_D$+[Ag])		
	Value	Error
AB$_{max}$	1816.7	190.41
k$_D$	51.282	20.192
Chisq	37041	NA
R	0.98723	NA

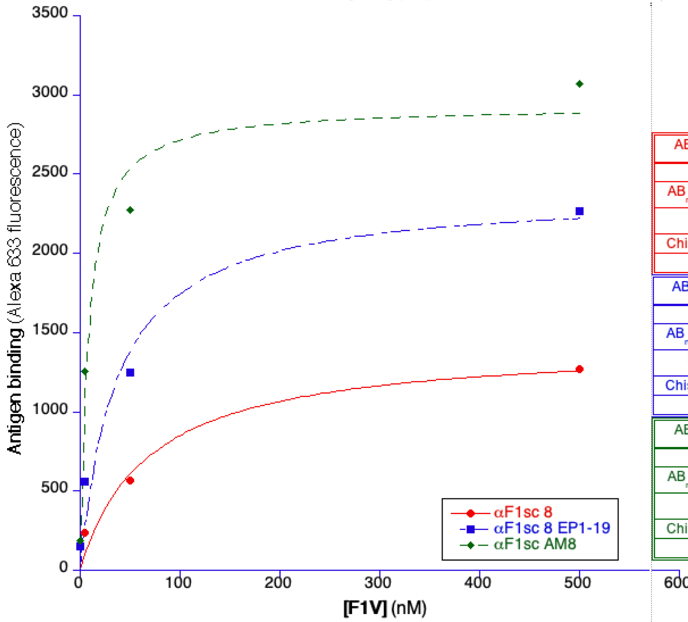

AB =AB$_{max}$*[Ag]/(k$_D$+[Ag])		
	Value	Error
AB$_{max}$	1429	237.88
k$_D$	67.708	40.398
Chisq	4.7814e+4	NA
R	0.96808	NA

AB = AB$_{max}$*[Ag]/(k$_D$+[Ag])		
	Value	Error
AB$_{max}$	2382.5	307.61
k$_D$	36.296	18.941
Chisq	1.1594e+5	NA
R	0.97716	NA

AB =AB$_{max}$*[Ag]/(k$_D$+[Ag])		
	Value	Error
AB$_{max}$	2928.7	245.45
k$_D$	7.8219	3.3762
Chisq	1.5067e+5	NA
R	0.98376	NA
