# Supplementary material for: Characterization of two affinity matured Anti-Yersinia pestis F1 human antibodies with medical countermeasure potential
Source: PLoS One. 2024 Jul 2;19(7):e0305034. doi: 10.1371/journal.pone.0305034 (PMC11218954; doi:10.1371/journal.pone.0305034)
Supplement: S2 Fig — (A) Setup of whole cell enzyme-linked immunosorbent assay (ELISA, top) and surface plasmon resonance (SPR) kinetic study. (B) ELISA data obtained for original anti-F1 IgGs (αF1Ig 2 and 8) or affinity matured (αF1Ig AM2 and 8) antibodies are shown. Experiments were performed in triplicate and each data point averaged. The standard deviation of each data point was calculated, and shown as the error bar. Data were fitted to the one-site specific binding equation. AB = antibody-F1 binding; ABmax = antibody-F1 binding at saturation; KD = dissociation constant = half saturating antibody concentration ([Ab]). (C) SPR sensograms obtained for each parental and affinity matured antibody are shown. The dissociation constant and the errors shown in Table 1 were averages and standard deviations of the KD values measured in these SPR experiments. (PDF) [file pone.0305034.s002.pdf]

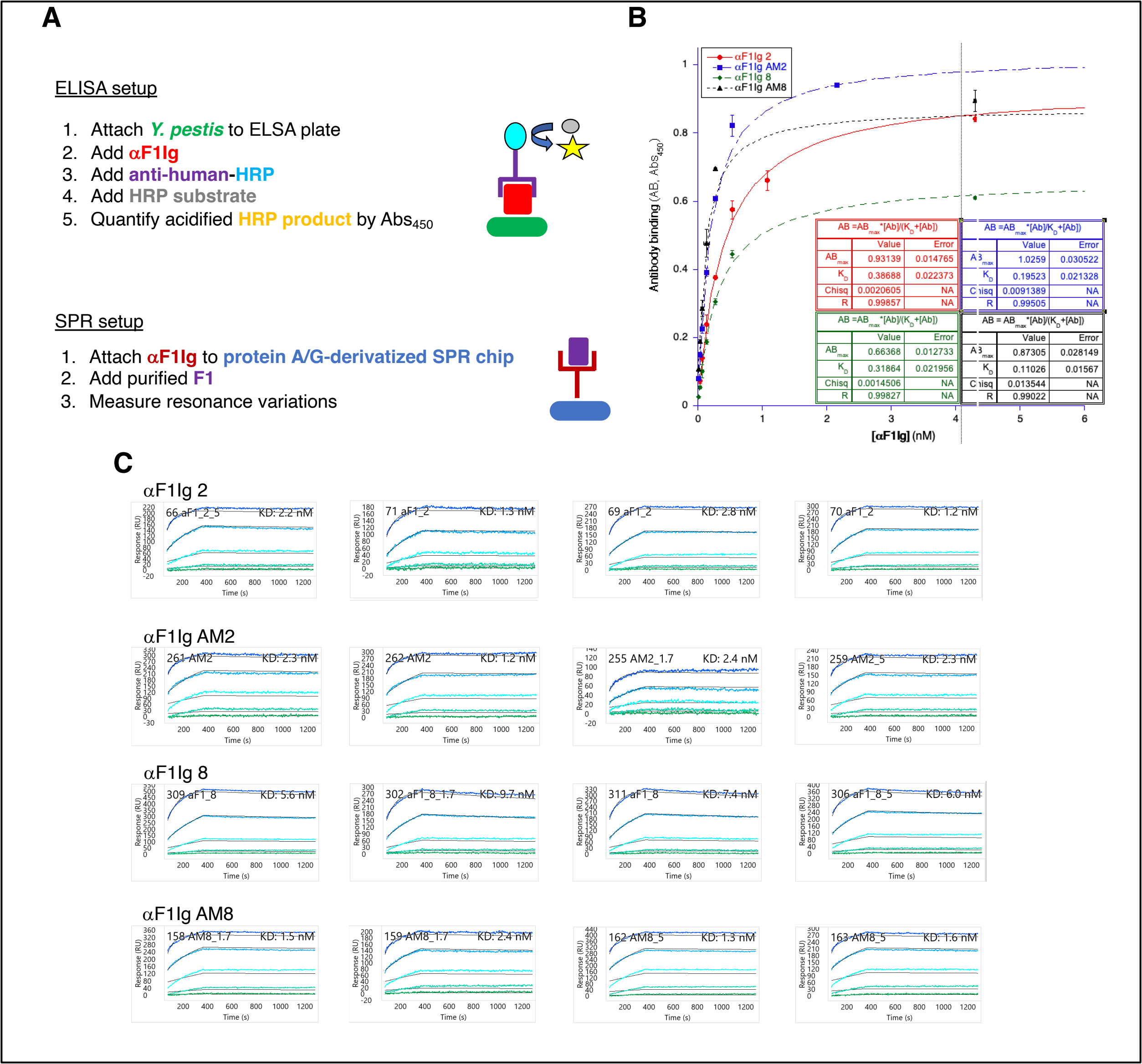
A
ELISA setup
1. Attach Y. pestis to ELSA plate
2. Add αF1Ig
3. Add anti-human-HRP
4. Add HRP substrate
5. Quantify acidified HRP product by Abs450
SPR setup
1. Attach αF1Ig to protein A/G-derivatized SPR chip
2. Add purified F1
3. Measure resonance variations
B
αF1Ig 2
αF1Ig AM2
αF1Ig 8
αF1Ig AM8
Antibody binding (AB, Abs450)
[αF1Ig] (nM)
AB =ABmax*[Ab]/(KD+[Ab])
Value Error
ABmax 0.93139 0.014765
KD 0.38688 0.022373
Chisq 0.0020605 NA
R 0.99857 NA
AB =ABmax*[Ab]/KD+[Ab])
Value Error
ABmax 1.0259 0.030522
KD 0.19523 0.021328
Chisq 0.0091389 NA
R 0.99505 NA
AB =ABmax*[Ab]/(KD+[Ab])
Value Error
ABmax 0.66368 0.012733
KD 0.31864 0.021956
Chisq 0.0014506 NA
R 0.99827 NA
AB = ABmax*[Ab]/(KD+[Ab])
Value Error
ABmax 0.87305 0.028149
KD 0.11026 0.01567
Chisq 0.013544 NA
R 0.99022 NA
C
αF1Ig 2
66 aF1_2_5 KD: 2.2 nM
71 aF1_2 KD: 1.3 nM
69 aF1_2 KD: 2.8 nM
70 aF1_2 KD: 1.2 nM
αF1Ig AM2
261 AM2 KD: 2.3 nM
262 AM2 KD: 1.2 nM
255 AM2_1.7 KD: 2.4 nM
259 AM2_5 KD: 2.3 nM
αF1Ig 8
309 aF1_8 KD: 5.6 nM
302 aF1_8_1.7 KD: 9.7 nM
311 aF1_8 KD: 7.4 nM
306 aF1_8_5 KD: 6.0 nM
αF1Ig AM8
158 AM8_1.7 KD: 1.5 nM
159 AM8_1.7 KD: 2.4 nM
162 AM8_5 KD: 1.3 nM
163 AM8_5 KD: 1.6 nM
Response (RU)
Time (s)
