# Supplementary material for: Characterization of two affinity matured Anti-Yersinia pestis F1 human antibodies with medical countermeasure potential
Source: PLoS One. 2024 Jul 2;19(7):e0305034. doi: 10.1371/journal.pone.0305034 (PMC11218954; doi:10.1371/journal.pone.0305034)
Supplement: S1 File — The amino acid sequence (one letter code) of single chain antibodies αF1sc 2 and 8 before affinity maturation and after various rounds of affinity maturations (AM) are shown. (PDF) [file pone.0305034.s003.pdf]

αF1sc 2

GAHADIRMTQSPSSLSASVGDRVTITC*RASQSISSYLN*WYQQKPGKAPKLLIY*ATSSLQS*GVPSRFSGSGSGTDFTLTISSLQPEDFATYYC*QQSYSTPSS*FGQGTKVDIKSGGSTITSYNVYYTKLSSSGTQVQLVESGGGLVKPGGSLRLSCAAS*GFTFSDYYMS*WIRQAPGKGLEWVS*YISSSGSIIYYADSVKG*RFTISRDNAKNSLYLQMNSLRAEDTAVYYCAK*EIRRHDAFDI*WGQGTMVTVSS

αF1sc 2 EP1-42

GAHADIRMTQSPSSLSASVGDRVTITC*RASQSISSYLN*WYQQKPGKAPKLLIY*ATSSLQS*GVPSRFSGSGSGTDFTLTISSLQPEDFATYYC*QQSYSTPSS*FGQGTKVDIKSGGSTITSYNVYYTKLSSSGTQVQLVESGGGLVKPGGSLRLSCAAS*GFTFSDYYMS*WIRQAPGKGLEWVS*YISSSGSIIYYADSVKG*RFTISRDNAKNSLYLQMNSLRAEDTAVYYCAK*EIRKHDAFDI*WGQGTMVTVSS

αF1sc 2 EP2-3

GAHADIRMTQSPSSLSASVGDRVTITC*RASQSISSYLN*WYQQKPGKAPKLLIY*ATSSLQS*GVPSRFSGSGSGTDFTLTISSLQPEDFATYYC*QQSYSTPSS*FGQGTKVDIKSGGSTITSYNVYYTKLSSSGTQVQLVESGGGLVKPGGSLRLSCAAS*GFTFSDYYMS*WIRQAPGKGLEWV*SYISGSGSIIYYADSVKG*RFTISRDNAKNSLYLQMNSLRAEDTAVYYCAK*EIRKHDAFDI*WGQGTMVTVSS

αF1sc 2 EP3 -18 (αF1sc AM2)

GAHADIRMTQSPSSLSASVGDRVTITC*RASRSISGYLN*WYQQKPGKAPKLLIY*ATSSLQS*GVPSRFSGSGSGTDFTLTISSLQPEDFATYYC*QQSYSTPSS*FGQGTKVDIKSGGSTITSYNVYYTKLSSSGTQVQLVESGGGLVKPGGSLRLSCAAS*GFTFSDYYMS*WIRQAPGKGLEWV*SYISGSGSIIYYADSVKG*RFTISRDNAKNSLYLQMNSLRAEDTAVYYCAK*EIRKHDAFDI*WGQGTMVTVSS

αF1sc 8

GAHAQPVLTQSPSVSVSPGQTASITC*SGDKLGDRYAS*WYQQKPGQSPVLVIY*QDTKRPS*GIPERFSGSNSGNTATLTISGTQAMDEADYYC*QSWDSSAAV*FGTGTKLTVLSGGSTITSYNVYYTKLSSSGTQVQLQQWGAGLLKPSETLSLTCAVY*GGSFSGYYWS*WIRQPPGKGLEWIG*EINHSGNTNYNPSLKS*RVTISVDTSKNQFSLQLNSVTPEDTAVYYCAR*TRGYFDL*WGRGTLVTVSS

αF1sc 8 EP1 -19

GAHAQPVLTQSPSVSVSPGQTASITC*SGDKLGGRYAS*WYQQKPGQSPVLVIY*QDTKRPS*GIPERFSGSNSGNTATLTISGTQAMDEADYYC*QSWDSSAAV*FGTGTKLTVLSGGSTITSYNVYYTKLSSSGTQVQLQQWGAGLLKPSETLSLTCAVY*GGSFSGYYWS*WIRQPPGKGLEWIG*EINHSGNTNYNPSLKS*RVTISVDTSKNQFSLQLNSVTPEDTAVYYCAR*TRGYFDL*WGRGTLVTVSS

αF1sc 8 EP2 -24 (αF1sc AM8)

GAHAQPVLTQSPSVSVSPGQTASITC*SGDKLGGRYAS*WYQQKPGQSPVLVIY*QDTKRPS*GIPERFSGSNSGNTATLTISGTQAMDEADYYC*QSWDSSAAV*FGTGTKLTVLSGGSTITSYNVYYTKLSSSGTQVQLQQWGAGLLKPSETLSLTCAVY*GGSFSGYYWS*WIRQPPGKGLEWIG*EINRSGNTNYNPSLKS*RVTISVDTSKNQFSLQLNSVTPEDTAVYYCAR*TRGYFDL*WGRGTLVTVSS

The complementarity determining regions (CDRs, Kabat definition) in the variable light (VL, shaded in yellow) and variable heavy (VH, shaded in green) regions are italicized and underlined. The linker sequence connecting the VL and VH is shaded in grey. Mutated positions are shown in green in the parental antibody sequences (αF1sc 2 or 8), and the replacing amino acids at these positions in the matured variants, are shown in red.
