# Supplementary material for: Characterization of two affinity matured Anti-Yersinia pestis F1 human antibodies with medical countermeasure potential
Source: PLoS One. 2024 Jul 2;19(7):e0305034. doi: 10.1371/journal.pone.0305034 (PMC11218954; doi:10.1371/journal.pone.0305034)
Supplement: Attachment — Submitted filename: rebuttal for PLOSone submission..docx [file pone.0305034.s008.docx]

**Response to Reviewer #1 comments to PLOS Pathogens submission PPATHOGENS-D-22-02157**

*Reviewer #1: In “Antibody Therapy for Plague – Characterization of Two Affinity Matured Anti-
Yersinia pestis F1 Human Antibodies with Therapeutic Potential”, Velappan et al describe the development and characterization of two affinity matured antibodies to better target Y. pestis. Convalescent serum and monoclonal antibodies are historically broadly useful in the treatment of infectious diseases, particularly in the absence of existing therapeutics like antibiotics. Despite general antibiotic sensitivity, ones targeting Yp could be useful in treatment of advanced disease. F1 is famously antigenic, and antibodies targeting it have an established efficacy in animal models of disease. The authors use standard methods and describe their process for the maturation, optimization, and humanization of these antibodies. These study is overall reasonably well written and well conducted, but is quite light on supporting data, analysis, and description of their technological and scientific advances. While it brings no novel insight in to Yp biology, pathogenesis, or the
host-pathogen interaction, it may be of interest to researchers of anti-bacterial antibodies.*

We thank the reviewer for acknowledging the quality of our study and its description in the manuscript. However, we respectfully disagree on lack of technological and scientific advances of our work. Due to the multi-drug resistance of *Y. pestis*, high-quality antibody cocktails designed for prophylactic use against this organism are very important for national security and public health preparedness, and as such they represent important scientific contribution. Additionally, most α*Y. pestis* antibodies described in literature are of murine origin, and we could only find one human anti-F1 antibody. We not only have 2 human antibodies never describe in literature, but they also target 2 different epitopes of the F1 antigen. The affinity maturation of these antibodies and the demonstration of their efficacy in vitro and in vivo supports their use as potential prophylactics and/or immunodiagnostics. On the technological advance front, we recognize that our work is not groundbreaking, however it does provide very detailed, and therefore easily reproducible, protocols for human antibody cocktail selection, maturation, and characterization. Such methodologies will become increasingly more important in the era of antimicrobial resistance and viral pandemics. We have re-written introduction and conclusion to bring forth all these points.

*Reviewer #1: The conclusions are brief (one paragraph, and no citations). This provide some summary of the paper, but no particular insights or broader perspective. Significantly more should be done to put this in the broader context of the literature to make clear what area of science is actually advanced by this study. Certainly, it is premature to suggest medical utility without tolerability, etc, studies (line 465).*

The PLOSOne version of the manuscript contains a revised conclusions section (currently, three paragraphs) that provides broader perspective on how our research fits into medical countermeasures against plague. While we agree that our mAbs are not quite ready for advanced development, we do not agree with the statement that *it is premature to suggest medical utility*. The development of medical countermeasures, particularly for biodefense related pathogens, is very much a long-term effort. The fact that these mAbs performed exceedingly well in both in vitro and in vivo assays reflects the potential for medical of these antibodies. Additionally, the BALB/c mouse pneumonic plague model is very sensitive, and for any previously untested medical countermeasure to offer significant protection is an important achievement that will aid in future advanced development if funding becomes available.

*Limitation of pre-exposure prophylaxis, limiting its therapeutic applications. Also, the required doses are not realistic, relative to other antibody therapies mentioned such as Obiltoxaximab (10.1111/cts.12433), or following standard mouse to human dose conversions (doi:10.4103/0976-0105.177703). If proposing use as a drug, as throughout, more should be done to test this.*

As implied in previous comment (“the BALB/c mouse pneumonic plague model is very sensitive”), the doses required in our animal studies might be higher than what is required for human treatment. Again, we agree that these antibodies are not ready for clinical development, we are also aware of the possibility that their dosage might be too high. Nevertheless, the data presented in this manuscript are good motivation for us (or others) to continue to improve these antibodies if new funding becomes available.
*Figure 5, the presentation is poor, and its no clear why it would be thought the antibodies are not opsonic. Human cells would be a better choice to show opsonization by a human antibody, there are many well-suited ones just as easy to use as RAWs, which would remove confounding variables. Appropriate controls, including isotype, blocking Fc/FcR, and blocking phagocytosis are need.*

The RAW cells were used because it was an existing assay that could screen for in vitro activity prior to down-selection of candidates to be tested in mice.  These assays are used as a correlate for the mouse model.  Additionally, the “gold-standard” mAbs at that time (an anti-F1 and anti-LcrV) were both mouse derived antibodies.  An assay using human cells and other controls described by the reviewer will be used as advanced development progresses, but they were beyond the scope of the first screening/characterizing efforts described within the manuscript.  Lastly, if a human antibody works well in mouse cells, then we are assuming it works even better with human cells, thus we are “underreporting” potential activity of the antibodies. Also, not sure why the reviewer is claiming we didn’t think they were opsonic.

*Reviewer #1: A few strains are used, A1122, CO92, an attenuated form, and in several points its not apparent which are used, the reason for the change, and whether there should be differences between them. This should be more clear.*

For LANL confirmation of antibody binding to F1 in the context of bacterial cell surface (i.e. in its natural form), we needed a readily available, LANL approved, non-virulent Y. pestis strain. Strain A1122 met all these requirements. This strain contains the plasmids pFra (encodes F1) and pPCP1 (only found in Y. pestis) but lacks the plasmid pCD1 (essential for virulence). For the USAMIRID in vitro macrophage assays, we needed an attenuated strain available in-house. We chose an excluded version of CO92 strain, which lacks the *pgm* locus (a 102 Kb locus responsible in part for iron acquisition) and was also cured of the pPst plasmid. The genetic modifications in A1122 and excluded CO92 strains make them suitable for use under BSL-2 conditions and not subject to select agent regulations. However, neither of these genetic modifications impact capsule production. This allowed us to perform statistically robust F1-antibody interaction studies (ELISA and opsonization) without the need for high containment, keeping costs low and decreasing risk by working at a lower biosafety level. For the USAMIRID animal studies a fully virulent strain was required for infection, hence the choice of CO92 strain. This information is fully available in the manuscript.

*Clarification needed (Line 435) Animal challenge reads as prepared as previously described (58,59), citing two papers, that have different methods, and also appear different from that described herein.*

We thank the reviewer for catching this inaccuracy. We corrected the mistake in the PLOSOne version of the manuscript.

*Figure 4 unreadable, and contains significant distracting extraneous information.*

We thank the reviewer for this feedback, we have revised and simplified figure 4 and moved the “extraneous” information to supplementary figure 2.

*Figure 1, the data is not clear and lost in the diagram, and connections between figure and [Ag] = 1 nM not clear*

We thank the reviewer for this feedback, we have addressed the clarity issue by revising and simplifying figure 1.
