# Supplementary material for: Characterization of two affinity matured Anti-Yersinia pestis F1 human antibodies with medical countermeasure potential
Source: PLoS One. 2024 Jul 2;19(7):e0305034. doi: 10.1371/journal.pone.0305034 (PMC11218954; doi:10.1371/journal.pone.0305034)
Supplement: Attachment — Submitted filename: Response to reviewers 031124.docx [file pone.0305034.s009.docx]

PONE-D-23-29661

Characterization of Two Affinity Matured Anti-Yersinia pestis F1 Human Antibodies with Medical Countermeasure Potential

PLOS ONE

We thank the editor of PLOS1 and the reviewers for their critical reading of our manuscript. The document below contains comments from the reviewers in black, our response in blue and specific text changes to the manuscript in green.

Reviewer #1: The authors present a study on the further development of anti-F1 monoclonal antibodies for use as countermeasures against *Yersinia pestis* infection. The concept of using F1 as a target is questionable because, while it is a strong immunogen, it is not a required virulence factor for the pathogen, and the general consensus is that weaponized strains will be F1negative as the F1 protein is a principal component of western vaccine development.

We thank the reviewer for the thorough review of this manuscript. We are well aware of the virulence associated with nonencapsulated strains of *Y. pestis* and our research continues to examine novel medical countermeasures to include both active and passive protection against F1-negative strains. However, in order to be prepared for all possible threats, we must be ready for all scenarios and we envision anti-F1 mAbs to be part of a combination or cocktail approach. To further emphasize the relevance of our antibodies, a patent has been granted recently (Synthetic anti-plague antibodies; Lillo AM, Velappan N, Dichosa AEL, Liley LM, Kozimor SA; USA 2023 Patent Number 11702465). This is the same rationale that resulted in a multi-antigen (or fusion protein) vaccine being developed in both the U.K. and U.S. Additionally, to date, natural infection with F1-negative strains is exceedingly rare, so an anti-F1 mAb would be quite applicable in a public-health context and with growing concerns for antimicrobial resistant strains. The reviewed version of the manuscript includes an additional paragraph and references to underscore the importance of the nonencapsulated strains (see below).

Additionally, the regions where naturally occurring antibiotic resistant strains are found are among the most resource limited areas in the world; areas where a very expensive technology, like a therapeutic monoclonal antibody could not be employed due to cost. These are items that really need to be discussed in the paper and are absent from it.

The antibodies described in our manuscript have good resistance to aggregation at high temperatures for extended periods of time. Since refrigeration is one of the major issues facing the developing world in utilizing monoclonal antibody therapy, highly thermostable antibodies such as described here should increase utility of therapeutic monoclonal antibodies. Issues such as socioeconomic constraints in various parts of the world that could limit the development of medical technologies and medical countermeasures, are outside of the scope of this manuscript. We believe this concept to be subjective commentary and is more political or policy-driven in nature.

Additional issues with the study are a general overestimation of the beneficial effects of their affinity maturation system, which is burdensome and had a negligible outcome on survival and affinity as determined by SPR, which is really the gold standard here. The writing in many areas is unclear, specific examples are given below. […] The affinity of the binding domain is only one small part of the functional capacity of an antibody, and it really doesn’t seem like the whole process here improved on antibody binding anything more marginally.

In vitro affinity maturation, the counterpart of somatic hyper mutation in B cells, is an integral part of antibody engineering (<https://www.ncbi.nlm.nih.gov/pmc/articles/PMC8726058/>). The error-prone PCR based affinity maturation we employed in our work allows for amino acid changes in both CDR regions as well as the more conserved framework regions. These changes have the potential to impact antibody binding affinity and stability, often in unpredictable ways. Considering the great potential value of the maturation process we found it important to employ it for this work, and to describe it at an unprecedented level of detail to the display technology community. We consider this a very valuable component of our paper.

In reference to the comment that the measured affinity improvements are minimal and that SPR is the gold standard assay that should count the most, please consider the following perspective. There are major differences in the antigen and antibody formats among the different assays performed for this work. The yeast display-based affinity measurements (and the maturation process) used F1V and scFv, SPR used F1 and IgG, and ELISA used *Y. pestis*-displayed F1V and IgG. We surmise that the difference in antigen formats, is the main culprit for the discrepancies in the affinity values and the observed low affinity increments (or lack-there-of). For example, it is entirely conceivable that during affinity maturation (where we used F1V as the target) we selected αF1sc 2 mutants with improved binding at the interphase of F1 and V antigens. The lack of V antigen in the monomeric F1 used in SPR might explain the higher *K*_D_ values (lower affinity) measured using this technique. Additionally, although we agree that SPR is the gold standard in affinity measurements, the ELISA used here reflects a more real-world scenario (antibody interacting with *Y. pestis*-displayed F1 antigen) than the SPR assay. So it could be argued that αF1Ig AM2, which based on SPR did not have higher affinity than αF1Ig 2 has indeed been affinity matured (2-fold increase in affinity).

While we acknowledge that the affinity increment of our matured antibodies compared to the parental ones is not as high as we had hoped for, please notice that increased manufacturability is an additional goal of the maturation process. High level of display on the yeast surface is generally a proxy for high stability (e.g. thermostability), foldability and low aggregation (all associated to good manufacturability). Manufacturability, together with affinity, is a feature enriched in the maturation process. Therefore, matured antibodies are likely to have higher stability than the parental antibodies. Our data show that we have indeed achieved at least higher stability. Although the stability of matured antibody was not measured side by side with parental antibodies a previous publication of ours (<https://pubmed.ncbi.nlm.nih.gov/33294421/>) suggests that parental antibodies start to degrade after 2.5 weeks storage at 37°C, whereas the matured antibodies (this paper) show no sign of aggregation after 150 days.

To reflect the points in this part of the rebuttal, we have amended the manuscript as follows:

**Lines 276-299**

AM2 *vs* αF1Ig 2. A major cause of this discrepancy could again be the difference in the antigen format used in the two assays. During flow-based kinetic studies (and the affinity maturation process) we used dimer-enriched F1V complex, whereas for SPR kinetics we used monomer-enriched F1. It is possible that αF1 antibody 2 binds at the intersection between F1 and V antigens and that the antibody structure changes during affinity maturation were tailored to the presence of V antigen. The lack of V protein in the antigen format used for the SPR assay (monomeric F1, not F1V) might explain why this assay failed to reveal affinity maturation. Also notice, that although SPR is the gold standard in affinity measurements, the ELISA used here reflects a more real-world scenario (i.e. antibody interacting with *Y. pestis*-displayed F1 antigen) than the SPR assay. Therefore, ELISA data should weigh more in the decision of whether αF1Ig AM2 has indeed been matured.

Based on these observations we could conclude that both matured IgGs have indeed higher affinity for F1 antigen than parental IgGs. We do recognize that the increment in affinity was not as high as expected, however please note that increased manufacturability is an additional goal of the maturation process. This is the reason why during maturation we sort yeast that are not only strong antigen binders but also strong displayers. High level of display on the yeast surface is generally a proxy for high stability (e.g. thermostability), foldability and low aggregation (all associated to manufacturability). Therefore, matured antibodies are likely to have higher stability than the parental antibodies. Although in this work the stability of matured antibodies was not measured side by side with the parental counterpart, we previously reported that αF1Ig 2 and 8 start to degrade after 2.5 weeks storage at 37°C, whereas matured αF1Ig AM2 and AM8 (this paper) show no sign of aggregation after 150 days.

In summary, we can safely conclude that our yeast display-based affinity maturation process was successful and resulted in improved variants of parental antibodies αF1Ig 2 and αF1Ig 8, i.e. αF1Ig AM2 and αF1Ig AM8.

The way they analyze their binding data by flow cytometry doesn’t not appear to be a good reflection of the ability of the antibody to bind to the antigen, but rather a measure of binding as a function of expression level. If the cell could express the antibody at a high rate, then it bound a lot of antigens. However, that doesn’t mean that an antibody expressed at a lower rate doesn’t have a higher affinity.

Determination of antibody affinity by yeast display and flow cytometry analysis is a widely accepted method to determine antibody affinity. One essential requirement is that antibody concentration (determined by number of yeast cells and average number of antibody copies on their surface) is well below antigen concentration. In this way antigen concentration can be assumed to be constant during the interaction with yeast. Additionally, antibody-antigen binding is normalized by antibody display, so there is no bias due to display (see below). While differences are observed between the affinities of yeast-displayed scFvs measured by flow cytometry and the corresponding IgGs measured by SPR, we disagree that flow cytometry does not provide a helpful indication of the affinity of the reported scFvs. Due to the spacing on the surface of yeast, in this assay the affinity of scFvs is less affected by avidity than measurements from a bivalent IgG format. This results in a lower observed affinity than a format with higher avidity, but it allows analysis of the intrinsic affinity of the scFv, which improves during our affinity maturation process. The improvement in affinity of individual scFvs tested results from an increased affinity in this format, and clones (original and AM) have similar levels of expression on yeast (for example display level (PE mean) for original YP8, EP1-19 and EP2-8 is 685,694 and 767 respectively). Similar MFI for display means that the increase in MFI for antigen binding is due to increased affinity and not due to increase in display levels. This type of observation has been previously reported (see references below).

- *Zupancic JM, Desai AA, Schardt JS, Pornnoppadol G, Makowski EK, Smith MD, Kennedy AA, Garcia de Mattos Barbosa M, Cascalho M, Lanigan TM, Tai AW, Tessier PM. Directed evolution of potent neutralizing nanobodies against SARS-CoV-2 using CDR-swapping mutagenesis. Cell Chem Biol. 2021 Sep 16;28(9):1379-1388.e7. doi: 10.1016/j.chembiol.2021.05.019. Epub 2021 Jun 24. PMID: 34171229; PMCID: PMC8223476*
- *Marcus WD, Lindsay SM, Sierks MR. Identification and repair of positive binding antibodies containing randomly generated amber codons from synthetic phage display libraries. Biotechnol Progr. 2006;22:919–22*
- *Simons JF, Lim YW, Carter KP, Wagner EK, Wayham N, Adler AS, Johnson DS. Affinity maturation of antibodies by combinatorial codon mutagenesis versus error-prone PCR. MAbs. 2020 Jan-Dec;12(1):1803646. doi: 10.1080/19420862.2020.1803646. PMID: 32744131; PMCID: PMC7531523.*

The expression level of the scFv in in yeast is irrelevant to antibody production because the CDRs are put into a different expression system later on for production of functional antibody.[…] Furthermore, the whole antibody production design is poorly explained in the paper. There are numerous systems that exist not where CDRs can be cloned into human IgGs that are optimized with glycosylation for Fc receptor interactions and increased uptake.

In the ATUM process of scFv conversion to IgG the entire VL and VH (not just the CDRs) are inserted in an IgG scaffold, so the IgG productivity WILL be influenced by the mutation process.

The metrics for defining what was considered good binding are not defined. It’s not really apparent that there were any. It seems that the investigators just drew a gate at the top of the dot plot cluster, but there doesn’t appear to be a defined cutoff.

As stated above, during the maturation process we sort yeast with the highest level of antigen binding and surface display. When the yeast population has an oblong shape, the right tip of the population contains the yeast of interest.

[….] All of this reduces enthusiasm for the manuscript. More specific issues are given below.

Due to all the above-mentioned factors, we strongly believe that our decision to undertake affinity maturation of the anti-F1 parental antibodies is highly justified and very relevant to research focusing developing antibody-based medical countermeasures.

The intro is wordy and can be shortened. There is a lot of superfluous information there. Mab therapy for COVID was relatively ineffectual unless given very early during infection, and efficacy dropped off through mutation of the spike protein. They did not play a significant role in the pandemic.

We feel that every element of the introduction ties to concepts described later in the paper. This is true of the discussion of the Covid antibodies as well. This particular example illustrates the concept of antibody cocktails (and need-there-of) and puts the use of antibody therapeutics in the context of a real work scenario. Of course, these are discussion points and not an endorsement of any particular product.

Furthermore, going on about massive mutations of a functional antigen not being likely to happen doesn’t really have relevance to the current study, as F1 is not an antigen required for virulence, and F1 negative strains cause natural human disease and are the most likely kind of strain to be used in a bioterrorist attack to get around vaccines that have been developed.

We are well aware of the concerns about nonencapsulated strains and have devoted much of our research to this concept, but we disagree with the reviewer on several points. F1-negative strains very rarely cause human infections. While F1- strains have been isolated in nature we are only aware of a single documented case of these strains causing human disease. Even this case (Winter, 1960) could have been the result of an infection with a “weakly” encapsulated strain as opposed to a truly nonencapsulated strain. Please see below for the information added to the manuscript regarding nonencapsulated strains.

Line 78, define F1V for the reader.

Thank you for the suggestion, we have edited the document to add F1V (F1 and V antigen of *Y. pestis*)

Line 118-120: What was considered a high signal, was there a specific MFI cutoff? Also, wouldn’t the PE signal be directly related to the Alexa 633 signal in any case where there is binding? MFI is a characteristic of the amount of fluorescence on a given particle, therefore by definition a high -633 signal would require a high PE signal. You shouldn’t have one without the other. In a perfect world you should have linearity between your 633 signal and your PE signal if the mutated antibody were binding to the F1 at saturation. Thus a clone that has higher expression would artificially be promoted as a clone with better binding, even if the actual affinity could be lower when those CDRs are put into an antibody. It’s also unclear to this reviewer why higher expression in a yeast system would be reflective of any characteristic for the antibody later on given that the variable regions were inserted into a cassette system later on? Perhaps expanded on this discussion a bit further.

Our experience and observations with yeast displayed scFvs suggest that scFvs with higher levels of display are more stable. However, the antigen binding (AF633) signal does not necessarily correlate directly with the display (PE) signal for library sorting, particularly in earlier rounds in which the library is more diverse. The sorted library will contain variants which may display at a reasonable level (high PE signal) but do not have desired affinity for antigen (undesirable AF633 signal). Vice versa, some clones may display on yeast at a lower copy number (lower PE signal) but bind strongly to antigen (high A633 signal). Since usage as medical countermeasures was one of the major objectives of developing anti-F1 antibodies, we chose to emphasize both expression level and binding signal during affinity maturation and sorting. We sorted top 2-5% of the population rather than a specific value for antigen binding (A633 value).

Figure 1 caption was amended as follows: ***3)*** The portion of the library with the highest level of yeast display (phycoerythrin -PE- fluorescence) and antigen binding (Alexa 633 fluorescence) is sorted by flow cytometry (2-5% of the entire yeast population). Sorting is repeated 3 to 4 times at progressively decreasing antigen concentrations.

Supplementary figure 1 should be in the paper with figure 2. Just showing the MFI in figure 2 alone doesn’t give great information because it is doesn’t account for differences in expression. The Y axis of figure 2 should be labelled MFI, not antigen binding.

The revised manuscript contains the suggested changes.

The specific binding equation in the methods and materials needs some additional clarification. Throughout this the authors refer to antigen binding as a fluorescence value (633 fluorescence), which presumably is MFI though this is not clearly stated. Not sure if it is the value calculated using this equation. In the equation, AB max is maximum antigen/antibody binding, but what is that? Is that the highest MFI at the highest concentration of antigen? Is that just the MFI from a particular experiment? [ab] = antibody concentration, but concentration of what antibody? You don’t know the concentration of the yeast antibody so it must be the MFI of the SV tag antibody, which is not concentration. This is all unclear. Kd apparent dissociation constant using Kaleidagraph software. Better description of how this is calculated should be provided. It also needs to be defined, exactly where this equation was used because it isn’t apparent.

We thank the reviewer for pointing out the lack of clarity of this portion of the manuscript, and for giving us the opportunity to clarify some concepts that were not properly explained. Additionally, the clarification process has allowed us to spot several typos. The edits to the text in question and their location are indicated below.

**Lines 445-457**

flow cytometry. Antigen-antibody binding was reported by the Alexa 633 fluorescence (medium fluorescence intensity, MFI; 635 excitation, 660/20 emission). This yeast-associated fluorescence derives from streptavidin-Alexa 633 conjugate, interacting with the yeast-bound biotinylated antigen. Antigen-antibody binding (Alexa 633 fluorescence MFI) at each concentration of antigen was normalized to the level of antibody display on yeast, reported by Phycoerythrin fluorescence (MFI, 561 excitation, 582/15 emission). The resulting data (antibody-antigen binding and corresponding antigen concentration) were fit to the one-site specific binding equation using GraphPad software.

AB = AB_max_*[Ag]/( K_D_ +[Ag])	Equation #1

In this equation AB is antigen-antibody binding (Alexa 633 MFI, variable), AB_max_ is the antigen-antibody binding (Alexa 633 MFI) at antibody-binding saturation (constant), [Ag] is the initial antigen concentration (variable), and *K*_D_ is the apparent antibody-antigen dissociation constant.

We have also modified Figure 2 as shown below:

**Figure 2**. **Comparison of original and affinity matured yeast-displayed scFv antibodies**. (**A**) The yeast-associated fluorescence after incubation with biotinylated F1V and staining with streptavidin-APC (allophycocyanin) was measured by flow cytometry. Mean fluorescence intensity values at antigen concentrations of 500, 50, and 5 nM, and corresponding standard deviations (error bars) are reported for the original (αF1sc 2 and 8) and the affinity matured (αF1sc AM2 and AM8) antibody clones. 500 nM biotinylated myoglobin was used as a negative control antigen. (**B** and **C**) The same data reported in **A** were fit in the one-site binding equation [AB = ABmax*[Ag]/(*K*_D_+[Ag])] and *K*_D_ resulting from the fit are indicated. The nomenclature EPX-Y in the legend denotes the error-prone library number (X) and the best clone number (Y) identified from this library. The best affinity matured αF1sc 2 clone (indicated for simplicity as αF1sc AM2 in **A**) was clone 18 from error-prone library 3 (αF1sc 2 EP3-18). The best affinity matured αF1sc 8 clone (indicated as αF1sc AM8 in **A**) was clone 24 from error-prone library 2 (αF1sc 8 EP2-24).

Line 159-161: ‘not the observed affinity improvements’ rather ‘the magnitude of the difference in the affinity measured’

Thank you for the suggestion, we have made the requested correction, and the sentence reads as follows.

**Lines 179-181**

antibodies were measured in the same way (i.e. they were subject to the same error), the magnitude of the difference in the affinity measured (*K*_D_ of parental antibody/*K*_D_ of matured antibodies) is reliable.

Line 198-202: I would suggest that the difference is likely native vs. recombinant fusion protein. This idea should be expanded upon.

We thank the reviewer for the opportunity to expand on this idea. The following paragraph was added to the revised manuscript.

**Lines 247-263**

due to different antibody formats (more below) and antigen formats. Specifically, soluble recombinant F1V antigen was used in the flow cytometry assays, and native F1V was used in ELISA. The recombinant F1V used for flow cytometry sorting is dimer-enriched antigen purified from *E. coli*. In this construct the signal peptide on the native F1 gene is removed and a dipeptide linker (EF) is introduced at junction between F1 and the V proteins. The resulting chimera is produced as soluble protein. Although this recombinant protein was designed to mimic the native protein, the required manipulations could have potentially altered the structure and stability of the proteins in subtle ways. During affinity maturation, we used the recombinant F1V fusion protein. It can be hypothesized that the increase in affinity might be due to additional contact points and/or structural adjustments occurring in the yeast displayed scFv to the presence of the V antigen in the chimera, contributed to the more pronounced changes in affinity. In vitro antibody selection and maturation technologies are highly sensitive to antigen formats, hence differences between recombinant fusion protein and native cell surface protein binding should be anticipated. Nevertheless, we can state that αF1Ig AM2 and αF1Ig AM8 both recognize native and recombinant F1 and that, based on ELISA, the affinity gain achieved by the scFv display-based maturation method seems to be preserved when the antibody format transitions from single chain to full length IgG.

Line 203-211: SPR is the gold standard for affinity measurement. This should come before the ELISA data.
We thought that it was important to first verify that the AM antibodies recognized the native F1 antigen on the bacterial cell surface, hence we chose to present the ELISA data first. SPR experiments were only considered upon verification of native F1 recognition by ELISA experiments. Therefore, our preference is to present the data in the current order.

Line 216: by phagocytes, not just macrophages.

Thank you for the suggestion, the word change has been completed.

Line 222-223: move to discussion. In addition, have you considered the possibility that your antibodies induced a higher rate of intracellular killing of the Yersinia compared to the control. That would also read out as reduced bacteria following cell lysis.
We have added a qualifier that this difference could be associated with an increased amount of intracellular killing.

Line 22. The positive control mouse-derived aF1 mAb F1-04-A-G1 appeared to be more potently opsonic than aF1Ig AM2 and aF1Ig AM8 or there is a possibility that AM2 and AM8 could potentiate better intracellular bacterial killing of the bacteria. However more work is required to understand the precise mechanism of action of these antibodies and the most likely explanation is that this difference in recovered bacteria at this early time point could be an artifact due to a better response of the mouse-derived RAW264.7 cells to a murine antibody (Figure 5).

237-239: You cannot extrapolate a human MaB therapy dose from mice, it’s not worth bothering doing so. They have different kinetics and metabolism of antibodies. Not only that, but you put a human antibody into a mouse, which will enhance it’s clearance. There are mab therapies that go into 150-200mgs in humans.
Thanks for the comments we have adjusted this sentence.

I’d much rather see survival curves than a table.

The number of experiments and experimental parameters are more clearly presented and described (including statistics) in tabular format. Experiment #1, all animals either lived or died with identical times-to-death, experiment #2 and #3 results are identical for survival with differences noted in TTD of the two animals receiving the human anti-F1 test mAb, and experiment #4 all mice succumbed to infection or were euthanized in accordance with early endpoint euthanasia with very few differences in TTD (ranging from 3-5 days). These data do not lend themselves to be effectively presented in graphical representation. The table gives detailed survival, TTD, and statistical analyses, however if making graphs is a prerequisite for publication we will comply.

Your whole paper lacks even a mention of F1 negative pestis. It is very relevant here, and really needs to be included. F1 is a great antigen, it is fully protective at very low doses in a single shot in animal studies but hasn’t gained approval because of the African Green Monkey studies at USAMRIID and the fact that it is completely ineffective against fully lethal F1 negative strains. Bioweapons will likely be F1 negative because it is a prominent vaccine target. The other issue is the fact that mAb therapy is out of reach, cost-wise in the regions where antibiotic resistant bacteria are present. (Madagascar/central Africa). That will greatly limit usage.

As stated above, the socioeconomic impact of an endemic region on monoclonal antibody research is outside the scope of this research manuscript. Additionally, the reason the F1-V vaccine has not progressed to FDA approval is more complicated than alluded by the reviewer. The F1-V vaccine is not completely ineffective against nonencapsulated strains, but the efficacy is reduced. The immune response to the LcrV component of the fusion protein subunit vaccine does offer some protection. Overall, the subunit vaccines are somewhat less effective in pneumonic plague compared to bubonic plague, in particular the GLP/GMP material compared to bench scale preparations. This has resulted in further work and further adjuvating of these plague vaccines. This topic is outside the scope of the manuscript, and we have added a description of the F1- strains to the revised discussion.

We have added the following paragraph and references to the discussion:

***Lines 351-*360**

Our efforts to develop novel anti-F1 mAbs will be beneficial to both the biodefense and public health research communities. While the overwhelming number of human plague cases have been caused by encapsulated strains of Y. pestis, nonencapsulated strains have been isolated in nature (Meka-Mechenko, 2003; Eppinger et al., 2012). However, very few documented cases of human disease initiated by these strains exist (Winter et al., 1960). Nevertheless, nonencapsulated strains remain virulent in animal models of plague and accordingly, other antigenic targets (e.g. LcrV) should always be considered when developing a medical countermeasure against plague (Drozdov et al., 1995; Friedlander et al., 1995; Welkos et al., 1995; Davis et al., 1996; Du and Wang, 2016; Biryukov et al., 2021; Biryukov et al. 2023). We envision an effective anti-F1 mAb to be beneficial in most cases of plague, but a cocktail approach would certainly provide the most complete protection against a number of different Y. pestis strains with differing antigen profiles (e.g. nonencapsulated).

References:

Eppinger, M., Radnedge, L., Andersen, G., Vietri, N., Severson, G., Mou, S., et al. (2012). Novel plasmids and resistance phenotypes in Yersinia pestis: unique plasmid inventory of strain Java 9 mediates high levels of arsenic resistance. PLoS One 7 (3), e32911. doi: 10.1371/journal.pone.0032911

Drozdov, I. G., Anisimov, A. P., Samoilova, S. V., Yezhov, I. N., Yeremin, S. A., Karlyshev, A. V., et al. (1995). Virulent non-capsulate Yersinia pestis variants

constructed by insertion mutagenesis. J. Med. Microbiol. 42, 264–268. doi: 10.1099/ 00222615-42-4-264

Friedlander, A. M., Welkos, S. L., Worsham, P. L., Andrews, G. P., Heath, D. G., Anderson, G. W. Jr., et al. (1995). Relationship between virulence and immunity as revealed in recent studies of the F1 capsule of Yersinia pestis. Clin. Infect. Dis. Suppl 2, S178–S181. doi: 10.1093/clinids/21.supplement_2.s178

Davis, K. J., Fritz, D. L., Pitt, M. L., Welkos, S. L., Worsham, P. L., and Friedlander, A. M. (1996). Pathology of experimental pneumonic plague produced by fraction 1- positive and fraction 1-negative Yersinia pestis in African green monkeys (Cercopithecus aethiops). Arch. Pathol. Lab. Med. 120 (2), 156–163.

Du, Z., and Wang, X. (2016). Pathology and pathogenesis of Yersinia pestis. Adv. Exp. Med. Biol. 918, 193–222. doi: 10.1007/978-94-024-0890-4_7

Biryukov, S., Dankmeyer, J. L., Shamsuddin, Z., Velez, I., Rill, N. O., Rosario- Acevedo, R., et al. (2021). Impact of Toll-Like Receptor-Specific Agonists on the Host Immune Response to the Yersinia pestis Plague rF1V Vaccine. Front. Immunol. 12, 726416. doi: 10.3389/fimmu.2021.726416

Meka-Mechenko, T. V. (2003). F1-negative natural Y. pestis strains. Adv. Exp. Med. Biol. 529, 379–81. doi: doi: 10.1007/0-306-48416-1_76

Biryukov SS, Klimko CP, Dankmeyer JL, Toothman RG, Shoe JL, Hunter M, Rill NO, Talyansky Y, Davies ML, Qiu J, Fetterer DP, Bozue JA, Welkos SL and Cote CK (2023) Live attenuated vaccines and layered defense strategies to combat infections caused by nonencapsulated Yersinia pestis. Front. Bacteriol. 2:1240698. doi: 10.3389/fbrio.2023.1240698

Winter, C. C., Cherry, W. B., and Moody, M. D. (1960). An unusual strain of Pasteurella pestis isolated from a fatal human case of plague. Bull. World Health Organ. 23 (2), 408–409.

It’s nice that you did the study showing that they are stable at 37C and 45C for long periods of time, but the overall impact of that finding is limited by the fact that they are serum proteins. IgG evolved through nature to exist at 37C for months.

We thank the reviewer for this comment. Antibodies indeed have evolved to be stable at 37°C within the context of serum. However, therapeutic antibodies are not stored nor developed in serum, and long-term stability is considered to be an important component of developability, which includes high expression levels, good solubility, and stability in storage (i.e. good manufacturability). [Jain et al, 2017] (*Jain 🡪 K Dane Wittrup PI)*

Further, differences in sequence and post-translational modifications between particular antibodies can create differences in the stability of those antibodies. [Nimmerjahn, et al. Nature Immunology (2023) ; vol 24, pp 1244-1255] Our intention for this stability study was to ensure that these two particular antibodies passed the stability test for manufacturability.

In order to thoroughly address the reviewer’s concerns, we have extensively modified the “Antibody stability measurements by DLS” section by restructuring the narrative and adding new text (in green)

**Lines 188-226***.*

Antibody stability measurements by DLS

Antibody drugs not only need to bind a target molecule, but they also need to be developable, i.e. they need to be expressed in high yield, have good solubility, be stable during long-term storage, and not be toxic. Although antibodies have evolved to be stable at 37ºC in serum, tracking antibody thermostability by dynamic light scattering (DLS) allows to assess antibody-specific shell life, but also propensity to aggregation, fragmentation, and potential generation of chemical by-products that could be toxic in vivo. Our DLS measurements were performed in the absence of preservatives (only saline solution) to assess the unaided resilience of our antibodies and shorten the duration of the experiment (stability at 37ºC) or avoid precipitation of adjuvants (high temperature stress). DLS has been used previously to demonstrate the structural integrity of monoclonal antibodies in solution [52]. DLS measurement of the hydrodynamic radius of both αF1Ig AM2 and αF1Ig AM8 in PBS before heat stress, revealed single peaks (100% of total species detected) corresponding to 12.12 and 14.91 nm radius, respectively. At 37°C, hydrodynamic radius measured consistently in the 10-17 nm range for both antibodies, as well as the control antibody, over the course of a 150-day incubation period (**Figure 3**). Possible aggregates (higher hydrodynamic radius) appeared at the 60^th^ day of incubation as peaks representing only 0.1% to 0.3% of the molecules detected. Since larger particles (aggregates) scatter light better than small particles, [53] these data suggest a negligible level of aggregation. We conclude that our anti-pestis antibodies could be stable for more than 150 days in harsh environments (37ºC is an extreme ambient temperature) when stored in the presence preservatives.

IgGs were also subjected to DLS temperature scan from 37 to 55°C. For αF1Ig AM2, temperatures and hydrodynamic radius sizes were as follows: 37°C = 17.1 nm, 45°C = 16.9 nm, and 55°C = no signal. Since the signal from the antibody disappeared somewhere between 45°C and 55°C, we decided to take more measurements between 45°C and 55°C to more accurately ascertain at what temperature the aggregation occurred. Unfortunately, due to constrains in antibody availability, we were only able to perform a more accurate assay with αF1Ig AM8. For this antibody the temperatures and sizes measured were: 37°C = 15.3 nm, 45°C = 15.7 nm, 47°C = 14.8 nm, 49°C = 14.6 nm, 51°C = 16.6 nm, 53°C = 6.7 nm, and 55°C = no signal. Lack of signal at 55°C suggests antibody precipitation out of solution at Tm = 55 for both antibodies. We concluded that at least αF1Ig AM8 is thermostable up to 51 °C. We were able to assess that aggregation and fragmentation at 0.3 μM antibody concentrations is negligible up to 45°C (*α*F1Ig 2) and 51°C (*α*F1Ig AM8). These thermostability data together with the high production yields (section above) suggest good manufacturability.

New references

Jain, et. al., “Biophysical properties of the clinical-stage antibody landscape”, PNAS (2017): vol 114, no 5, pp 944-949. [www.pnas.org/cgi/doi/10.1073/pnas.1616408114](http://www.pnas.org/cgi/doi/10.1073/pnas.1616408114)

Line 271: The technology is academically interesting, but the drawbacks haven’t been fully highlighted. It is labor intensive, and of the antibodies generated one showed no appreciable increase in affinity via SPR, the gold standard, and the one that did show an appreciable increase, worked worse in vivo. That’s not a great selling point.

This comment is addressed throughout this rebuttal and in the edited version of the manuscript.

Reviewer #2: This manuscript describes the maturation and characterization of mAbs as a potential therapeutic for pneumonic plague. As Y. pestis is categorized as a Category A agent, continuing research into mechanisms of pathogenicity as well as new treatment strategies is important to our defense.
Overall, the manuscript was clear. Please find my suggestions below, organized by Line:

We thank the reviewer for a through and critical review of our manuscript.

87- replace ] with ) after technologies

Thank you, the correction has been made.

172 – This sentence describes the direct output of your DLS measurements, specifically single peaks. However, that is not what is shown in Figure 3, which is referenced. Either consider adding your DLS scan output or move the reference to Figure 3 to the end of the sentence in Line 174.

We thank Reveiwer #2 for pointing out this discrepancy. The relevant portion of the manuscript now reads:

**Lines 179-181** At 37°C, hydrodynamic radius measured consistently in the 10-17 nm range for both antibodies, as well as the control antibody, over the course of a 150-day incubation period (**Figure 3**).

179-180 – Why weren’t both IgGs subjected to the same DLS temperature scan? AM2 was analyzed at only 3 temperatures, whereas AM8 was analyzed at 7.

We thank Reviewer #2 for pointing out this difference in measurement strategy. For our first DLS temperature scan, performed on antibody aF1Ig AM2, we chose to measure at 37°C, 45°C, and 55°C. Since the signal from this antibody disappeared somewhere between 45°C and 55C, we decided to take more measurements between 45°C and 55°C for antibody aF1Ig AM8 than we had with aF1Ig AM2. We chose to add measurements at 47, 49, 51, and 53°C to fill in the gap. Since the signal for aF1Ig AM8 was stable up through 51°C, started to drop at 53°C, and disappeared at 55°C, we surmised that both antibodies are stable up to 50°C, and did not think it necessary to repeat the added measurements for aF1Ig AM2. The relevant portion of the manuscript now reads:

**Lines 187-192** For αF1Ig AM2, temperatures and hydrodynamic radius sizes were as follows: 37°C = 17.1 nm, 45°C = 16.9 nm, and 55°C = no signal. Since the signal from the antibody disappeared somewhere between 45°C and 55°C, we decided to take more measurements between 45ºC and 55ºC to more accurately ascertain at what temperature the aggregation occurred. Unfortunately, due to constrains in antibody availability we were only able to perform a more accurate assay with αF1Ig AM8.

220 – There is a random e after the word either.

Thank you, the correction has been made.

225 – Language should be modified for publication. Hours should also be spelled out not abbreviated h.

Thank you for suggestion, the corrections have been made.

Conclusion – In general, the conclusion could be strengthened with specific result details and references that support why they are favorable findings. It is a bit bold to declare dose dependency of your mAb protection when your LD50s vary significantly across your experiments.

We agree with the reviewer and unfortunately the inherent difficulty associated with aerosol delivery and reproducibility did not allow for more precise comparisons. However, our data do trend towards demonstrating dose-dependency. We have altered the description in the discussion to read:

***Line 371-375***

post-exposure or prophylactic use. Our in vivo studies demonstrated a significant protective effect when delivered prior to exposure. This protection in the mouse mode of pneumonic plague appears to be dose-dependent. However, due to the inherent variability associated with small-particle aerosol delivery of *Y. pestis* resulting in different doses of delivered bacteria, dose-dependence is not strongly supported. Further, these antibodies have favorable

Figure 2 – Does this data represent combined experiments (if so how many) or is it representative (if so of how many)?

Representative data from three experiments performed with different batches of biotinylated F1V is given in Figure 2. The dimeric F1V (the only purified protein available to us at the start of the project), was very unstable and required small batch biotinylation. At the recommendation of reviewer 1, we have added graphs showing data fitting and calculated *K*_D_s to Figure 2 to provide additional explanation on how the data in the graph were obtained.

Figure 3 – Your methods state that the hydrodynamic radius of a control antibody was also measured. That should be included in figure 3 to demonstrate the variability in your IgGs over time is standard or better than standard.

The data has been added to Figure 3 and the updated figure is given below.

**Figure 3**. **Antibody stability by dynamic light scattering analysis**. The hydrodynamic radius of anti-F1 affinity matured antibody 2 and 8 (aF1Ig AM2, orange; aF1Ig AM8, green) was ~14 nm and did not change after 150 days storage at 37°C in PBS. These results indicate lack of protein aggregation in the conditions tested. A mouse anti-lysozyme IgG stored at 4°C for the duration of the experiment was used as a control (blue). In this figure, two per moving average trendline is indicated as dotted lines.

Figure 5 – Statistical stars should be centered above data.

Thank you, the correction has been made.
